# Adrenaline, Takotsubo Cardiomyopathy, Anaphylaxis, and Kounis Syndrome (ATAK) Complex: Clinical Phenotypes, Differential Diagnosis, and Management

**DOI:** 10.1016/j.jaccas.2026.108345

**Published:** 2026-05-14

**Authors:** Kristian Galanti, Elena Bacigalupi, Marco Zimarino, Antonino Scarinci, Sabina Gallina, Monica De Gaspari, Stefania Rizzo, Cristina Basso, Fabrizio Ricci, Vincenzo Cicchitti

**Affiliations:** a Department of Neuroscience, Imaging and Clinical Sciences, G. D'Annunzio University of Chieti-Pescara, Chieti, Italy; b Center for Inherited Cardiovascular Diseases, Genomic and Precision Medicine, WellSpan Health, York, Pennsylvania, USA; c Department of Cardiology, Policlinico Casilino, Rome, Italy; d Department of Cardiology, Policlinico SS. Annunziata, Chieti, Italy; e University Cardiology Division, Heart Department, Policlinico SS. Annunziata, Chieti, Italy; f Institute for Advanced Biomedical Technologies, G. D'Annunzio University of Chieti-Pescara, Chieti, Italy; g Department of Cardiac, Thoracic. Vascular Sciences and Public Health, Cardiovascular Pathology Unit, University of Padova, Padova, Italy

**Keywords:** ATAK, endomyocardial biopsy, eosinophilic myocarditis, Kounis syndrome, multimodality cardiovascular imaging, Takotsubo cardiomyopathy

## Abstract

**Background:**

Anaphylaxis is a severe hypersensitivity reaction that may involve the heart through overlapping inflammatory, vascular, and catecholaminergic mechanisms. The ATAK (adrenaline, Takotsubo cardiomyopathy, anaphylaxis, Kounis syndrome) complex describes a clinical continuum in which hypersensitivity-mediated inflammation and endogenous or exogenous catecholamines promote endothelial injury, coronary vasomotor and microvascular dysfunction, and myocardial damage.

**Case Summary:**

A 79-year-old man with urinary sepsis developed an antibiotic-related hypersensitivity reaction followed by acute pulmonary edema with severe biventricular dysfunction. Multimodality cardiovascular imaging and early endomyocardial biopsy assessment supported a diagnosis of Kounis syndrome with superimposed Takotsubo cardiomyopathy, consistent with the ATAK complex. Recovery followed administration of high-dose corticosteroids and intensive supportive care.

**Take-Home Messages:**

Increasing evidence suggests an association between anaphylaxis and cardiac involvement, although mechanisms remain incompletely understood. Catecholamine excess may exacerbate myocardial injury and promote ATAK overlap phenotypes. Multimodality imaging and early endomyocardial biopsy can refine diagnosis and guide targeted management.

## Visual Summary

**Figure:**
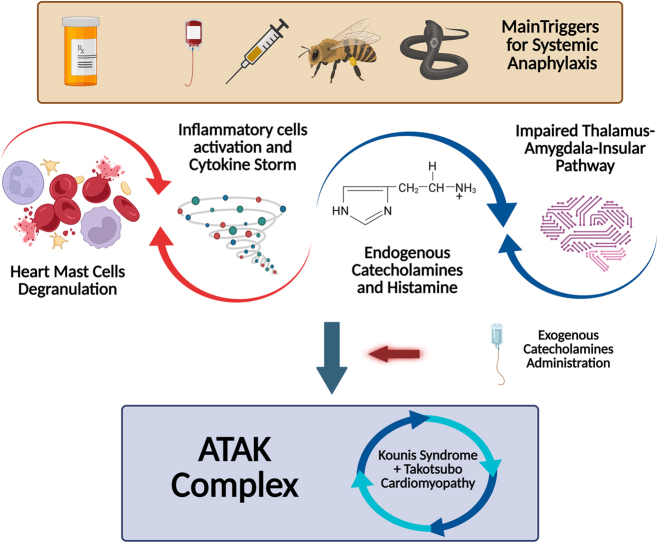

## Case Presentation

A 79-year-old man presented to the emergency department with a 2-day history of fever and dysuria. His medical history was notable for hypertension, non–insulin dependent diabetes mellitus, chronic eczema, postsurgical hypothyroidism, and pulmonary emphysema. On admission, he was diagnosed with sepsis, most likely of urinary origin, and empiric antibiotic therapy with piperacillin/tazobactam (4.5 g three times a day) was initiated. Shortly thereafter, he developed a diffuse erythematous rash, which resolved after treatment with corticosteroids and antihistamines. Only subsequently did the patient report a previous allergic reaction to amoxicillin/clavulanic acid that had occurred several months earlier, characterized by a cutaneous rash and facial swelling. As a result, antimicrobial therapy was promptly switched to ciprofloxacin (400 mg twice daily).

**Take-Home Messages**

• Increasing evidence suggests an association between anaphylaxis and cardiac involvement, although mechanisms remain incompletely understood.
• Catecholamine excess may exacerbate myocardial injury and promote ATAK overlap phenotypes.
• Multimodality imaging and early endomyocardial biopsy can refine diagnosis and guide targeted management.

**Visual Summary:**
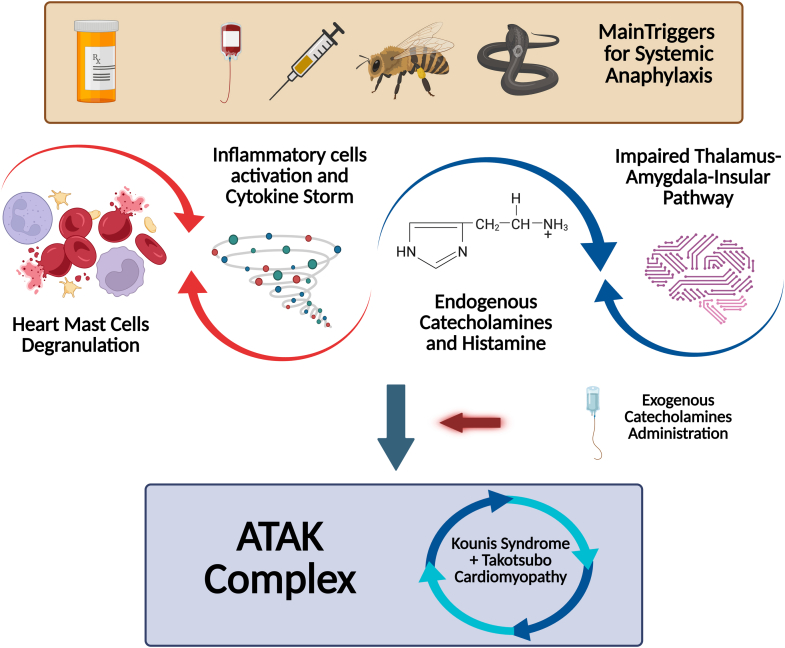
Proposed Pathophysiological Pathway for the ATAK Complex An allergic reaction, through the activation of inflammatory cells, initially prompts a Kounis syndrome phase with associated endogenous catecholamine, cytokines, and histamine release. The concomitant physical stress induced by systemic anaphylaxis and the administration of additional exogenous catecholamines to counteract the shock may serve as a trigger for Takotsubo cardiomyopathy. The combined effect of these 2 pathways could lead to the ATAK (adrenaline, Takotsubo cardiomyopathy, anaphylaxis, Kounis syndrome) complex.

During hospitalization, the patient developed progressively worsening dyspnea, which culminated 3 days after admission in acute pulmonary edema and the new onset of a dilated hypokinetic cardiomyopathy. Given his rapid clinical deterioration, he required transfer to the intensive care unit for escalation of care. Coronary angiography showed unobstructed coronary arteries (Figure 1), and electrocardiography revealed sinus tachycardia with mild left ventricular conduction delay (Figure 2). Cardiac imaging revealed severe left ventricular dysfunction with apical hypokinesia. Initial management included high-dose methylprednisolone (1 g once daily), noradrenaline (0.3 μg/kg/min), and furosemide (250 mg, continuous infusion at 10 mg/h).

**Figure 1:**
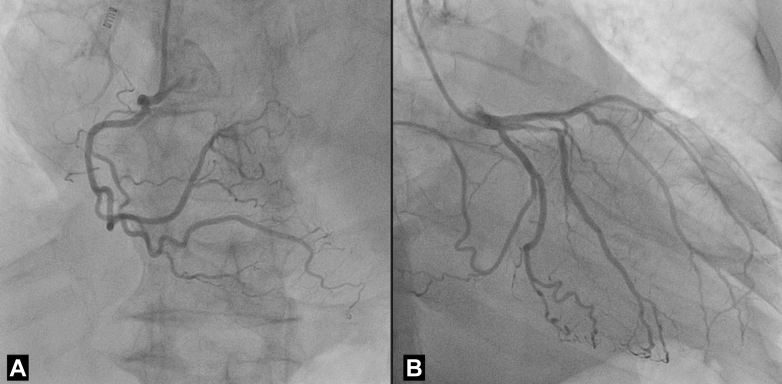
Invasive Coronary Angiography Showing Patent Epicardial Coronary Arteries (A) Left anterior oblique projection. (B) Right anterior oblique caudal projection.

**Figure 2:**
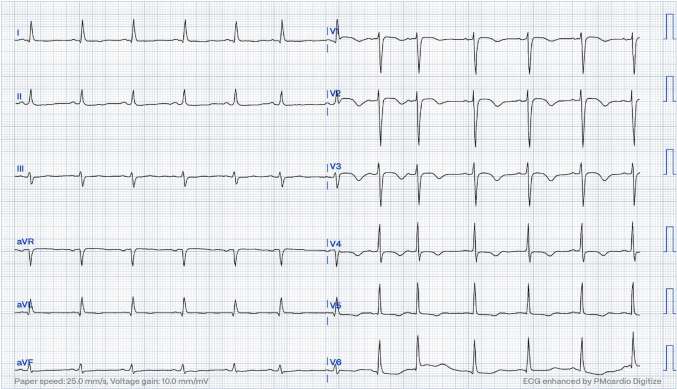
Electrocardiogram Showing Sinus Rhythm With No Atrioventricular Conduction Abnormalities, Single Supraventricular Ectopic Beat and Mild Left Ventricular Conduction Delay With Aspecific Ventricular Repolarization Abnormalities

The patient was transferred to the intensive care unit because of hemodynamic instability associated with acute biventricular failure, requiring advanced hemodynamic monitoring and continuous noradrenaline infusion to ensure adequate organ perfusion. Sedation was achieved with propofol and remifentanil. Intensive supportive management included continuous furosemide infusion, correction of electrolyte abnormalities, corticosteroid therapy, and a comprehensive diagnostic reassessment with microbiological sampling and infectious disease consultation. The patient required mechanical ventilation with a lung-protective strategy and was progressively weaned via pressure support to noninvasive ventilation. Clinical stabilization allowed subsequent transfer to the cardiac intensive care unit. Ten days later, repeat echocardiography (Figure 3) demonstrated partial improvement of left ventricular systolic function (left ventricular ejection fraction [LVEF]: 42%). Cardiac magnetic resonance revealed apical edema and nonischemic late gadolinium enhancement of the basal septum (Figure 4). Laboratory findings showed a marked elevation of troponin I (>10,000 pg/mL), associated with mild to moderate increases in creatine kinase (820 U/L) and creatine kinase–MB (68 U/L), along with peripheral hypereosinophilia (2.72 × 10^3^/μL). Based on the integrated findings, high-dose corticosteroid therapy was administered. Rheumatological tests were negative. An endomyocardial biopsy (EMB) revealed focal vasculitis and lymphomonocytic infiltration, with no eosinophils or myocardial necrosis (Figure 5). One month later, the patient was asymptomatic, with near-complete recovery of LVEF (54%) and resolution of the cutaneous rash. In light of the clinical course, underlying allergic predisposition, and integrated multimodal findings, this case supports a pathophysiological sequence beginning with a type I Kounis syndrome, followed by catecholamine-triggered Takotsubo cardiomyopathy, consistent with ATAK (adrenaline, Takotsubo cardiomyopathy, anaphylaxis, Kounis syndrome) complex.

**Figure 3:**
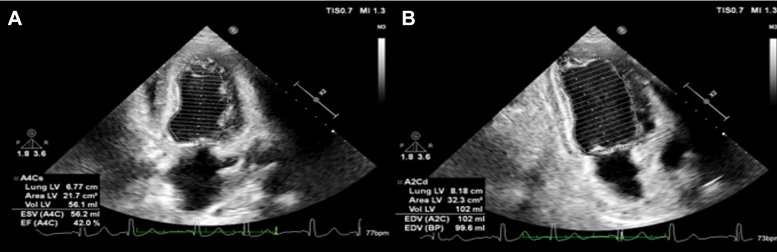
Repeat Transthoracic Echocardiography Revealing Improvement of Left Ventricular Systolic Dysfunction (A and B) Two-dimensional transthoracic echocardiogram revealed mild left ventricular systolic dysfunction (ejection fraction: 45%-46%) with hypokinesia of the basal inferior interventricular septum and apex.

**Figure 4:**
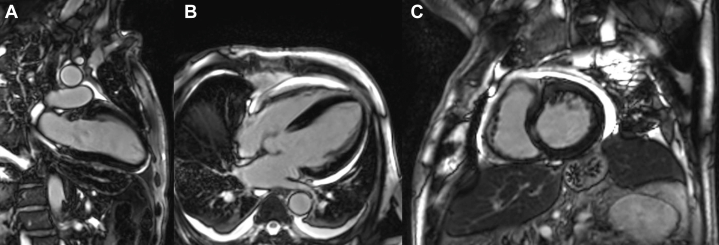
Cardiac Magnetic Resonance Showing Presence of Late Gadolinium Enhancement (A) 2-chamber view. (B) 4-chamber view. (C) short-axis view. CMR showed midwall late gadolinium enhancement of the basal interventricular septum.

**Figure 5:**
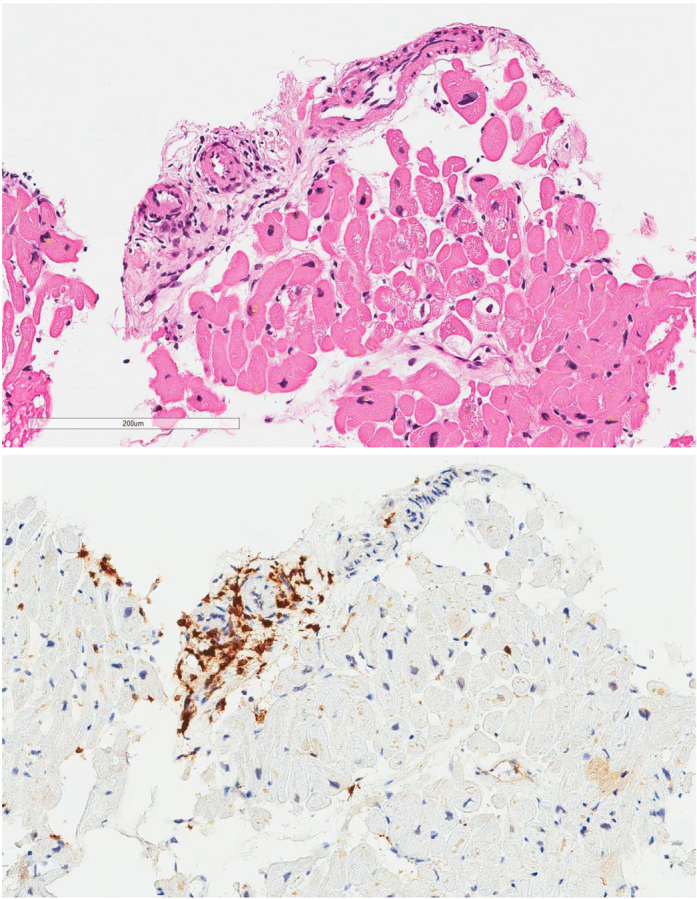
Endomyocardial Biopsy Showing Revealing Focal Vasculitis Abnormalities Without Eosinophilic Infiltration Molecular pathology investigation was found negative for adenovirus, cytomegalovirus, Epstein Barr virus, human herpes virus 6, herpes simplex virus, and parvovirus B-19 both in the endomyocardial biopsy samples and in the blood.

## Background

Anaphylaxis is a severe, life-threatening systemic hypersensitivity reaction whose epidemiology and clinical recognition have evolved over the past century.^1^ Despite growing awareness, its true global burden remains uncertain because of heterogeneous diagnostic criteria, variable study designs, and under-reporting.^2^^,^^3^ Available data suggest an annual incidence increase of approximately 7.4%, corresponding to 46 cases per 100,000 individuals.^2^ This rise is likely driven by environmental changes, increased allergen exposure, improved recognition, and more consistent reporting systems.^4^ Early clinical manifestations typically include cutaneous symptoms, such as urticaria and angioedema, and respiratory compromise, including laryngeal edema and bronchospasm.^5^ In severe cases however, cardiovascular involvement may predominate and critically influence prognosis.^6^ Mechanistically, allergen binding to immunoglobulin E on high-affinity FcεRI receptors induces mast cell and basophil activation,^7^ leading to rapid release of preformed mediators (eg, histamine, tryptase, chymase) and newly synthesized cytokines (eg, TNF-α, IL-4/6, IL-13), which drive the systemic response.^8^ Cardiac mast cells display distinct biological features, including strategic localization between myocardial fibers, within atherosclerotic plaques, and along coronary vessels.^9^ Upon activation, they release higher levels of chymase and renin compared with mast cells in other tissues.^10^ Renin, through AT1 receptor activation on sympathetic nerve endings, enhances local norepinephrine release,^11^ contributing to adrenergic overactivation and myocardial dysfunction. Cardiovascular involvement is further amplified in patients with pre-existing coronary artery disease. In this context, mast cells are more abundant and exhibit increased mediator release. In addition, commonly prescribed therapies, such as beta-blockers, may attenuate the response to first-line treatment with norepinephrine.^12^ This complex interplay between allergic, cardiovascular, and pharmacological factors underscores the need for prompt recognition and tailored management of anaphylaxis with cardiac involvement.

## Mini-Review

### Anaphylaxis-induced cardiac involvement: clinical phenotypes

Advances in our understanding of the inflammatory and hemodynamic consequences of anaphylaxis has shed light on the diversity of its cardiac manifestations.^12^ Here, we present the 3 main cardiac manifestations that may arise after an anaphylactic trigger. It is important to note that, while these conditions represent distinct nosological entities, the interconnection between some of them may explain the pathophysiological mechanism known as ATAK syndrome. Understanding this interaction is crucial to improve the management and prognosis of patients with anaphylaxis, especially those with concurrent coronary artery disease or heightened susceptibility to allergic reactions.

### Takotsubo cardiomyopathy

Takotsubo cardiomyopathy (TTC) is an acute, reversible syndrome characterized by regional left ventricular dysfunction, often mimicking acute coronary syndrome (ACS), yet occurring in the absence of obstructive coronary artery disease.^13^ It is often triggered by intense emotional or physical stress and predominantly affects postmenopausal women.^14^ As initial clinical suspicion typically arises from ischemic symptoms—chest pain, ECG changes (eg, T-wave inversion, QT prolongation), and elevated natriuretic peptides—coronary angiography is essential to exclude epicardial obstruction as the underlying cause of systolic dysfunction, thereby supporting the diagnosis. The classical pathophysiological framework of TTC emphasizes a catecholamine surge triggering myocardial stunning via β-adrenergic signaling. However, this catecholaminergic model alone fails to explain interindividual variability in susceptibility and outcomes. The variability likely reflects complex interactions between neurohormonal reactivity, hypothalamic-pituitary-adrenal axis regulation, and individual myocardial vulnerability.^15^ Functional neuroimaging studies demonstrate structural and connectivity differences in limbic and autonomic regulatory brain regions—thalamus, amygdala, basal ganglia—among TTC patients, highlighting altered stress processing as a potential predisposition.^16^ More recently, a distinct inflammatory vascular model has gained attention. TTC appears to begin with an acute coronary vascular insult involving glycocalyx shedding, endothelial barrier disruption, and microvascular spasm.^17^ This early process is driven by catecholamine-induced oxidative and nitrosative stress, which promotes superoxide and nitric oxide interaction, generating peroxynitrite and precipitating microvascular injury. The resulting coronary slow flow correlates with early left ventricular dysfunction and elevated natriuretic peptides, serving as an early marker of microvascular inflammation. In murine models, TTC is associated with a proinflammatory M1 macrophage response, without the typical reparative M2 shift seen after myocardial infarction. This sustained inflammatory state may explain why, despite apparent ejection fraction recovery, many patients experience prolonged exertional dyspnea, myocardial strain, and energetic impairment, consistent with a subacute myocarditis-like phase.^18^ In some cases, patchy fibrosis and long-term fatigue may develop, marking a third, chronic phase of the disease. This sequence—from acute coronary vasculitis to prolonged inflammation and potential fibrosis—recasts TTC as an inflammatory cardiomyopathy initiated by microvascular injury. It also provides a unifying framework for overlapping conditions such as stress-induced myocardial dysfunction during allergic reactions and the broader ATAK complex. Recognition of the vascular-inflammatory axis of TTC may stimulate novel therapeutic strategies aimed at mitigating microvascular inflammation and accelerating myocardial recovery.

### Kounis syndrome

Kounis syndrome (KS) is a form of ACS that arises during allergic or hypersensitivity reactions, where activated mast cells interact with inflammatory immune cells, leading to multiorgan involvement.^19^ Although KS can occur at any age, the peak prevalence (68%) is observed between the ages of 40 and 70. Predisposition to allergic disease and a history of prior hypersensitivity reactions may coexist with, and add to, traditional cardiovascular risk factors, including smoking, diabetes, and dyslipidemia. Diagnosis is based on clinical signs and symptoms, along with electrocardiographic and angiographic findings, which must be correlated with the patient's allergic history. Approximately 25% of patients with KS have a documented history of allergies, often linked to a specific trigger.^20^ Cardiac involvement is supported by the observation of a higher number of degranulated mast cells at sites of plaque erosion or rupture compared to surrounding areas. This can manifest in one of 4 pathological presentations: coronary vasospasm in individuals with normal coronary arteries (type I), coronary thrombosis in patients with inactive pre-existing atherosclerosis (type II), stent thrombosis (type III), or coronary artery bypass graft thrombosis (type IV).^21^ Key mediators released during KS include histamine, chymase, and tryptase, which contribute to vasoconstriction, platelet activation, and a drop in primarily diastolic blood pressure.^22^ In the clinical management of suspected KS, measuring serum tryptase levels may aid in distinguishing it from other forms of ACS. However, owing to the short plasma half-life of tryptase, it should be measured at initial patient presentation for reliable results.^23^

### The ATAK complex

First coined by Nicholas Kounis and formalized in 2016,^24^ the ATAK complex represents a pathophysiological combination of conditions unified by an allergenic trigger and fueled by the common denominator of catecholamines, mainly of exogenous origin.^25^ The synergistic effects of allergic inflammation, catecholamine toxicity, and coronary dysfunction converge to produce a highly complex clinical picture that remains under-recognized in routine practice. Currently, most of the available evidence regarding the ATAK complex is derived from case reports and case series (Table 1),^26^, ^27^, ^28^, ^29^, ^30^, ^31^, ^32^, ^33^, ^34^ highlighting the uncertainty surrounding its precise epidemiological, pathophysiological, and prognostic characteristics. Notably, the known triggers for this complex are varied, ranging from the administration of antibiotics^32^ to antineoplastic therapy sessions,^28^ vaccination,^31^ or the use of exogenous catecholamines.^27^ This broad spectrum of triggers increases the complexity of ATAK complex diagnosis and management, further emphasizing the need for comprehensive clinical knowledge and research to better define and understand this multifaceted syndrome.

**Table 1:** Summary of ATAK Complex Literature

First Author	Age/Sex	Trigger	Phenotype	Troponin Release	Imaging	Treatment
Singh et al^26^	48 y/male	Pituitary adenoma	TTC	No	No CAD, no RWMA	Fluids, steroids, inotropes
Gicquel-Schlemmer et al^27^	48 y/female	Epinephrine	TTC	No	Apical ballooning	Epinephrine, thrombolysis
Mustehsan et al^28^	60 y/female	Chemotherapy	KS and TTC	Yes	Apical akinesia	Epinephrine, steroids
Margonato et al^29^	60 y/female	Plasma-expander	KS and TTC	Yes	Apical ballooning, LGE	Steroids, HF therapy
Ballesteros et al^30^	50 y/male	Epinephrine	ATAK	Yes	Midventricular akinesia	Epinephrine, steroids
Minciullo et al^31^	54 y/female	SARS-CoV-2 vaccine	ATAK	No	Reduced LVEF	Epinephrine, steroids
Alarcón Gallardo et al^32^	77 y/female	Amoxicillin	ATAK	Yes	Apical akinesia	Epinephrine, steroids
Li et al^33^	67 y/male	Moxalactam	ATAK	Yes	No CAD, apical ballooning	Epinephrine, steroids
Puri et al^34^	63 y/female	Chemotherapy	ATAK	Yes	Reduced LVEF, RWMA	Epinephrine, steroids

ATAK = adrenaline, Takotsubo cardiomyopathy, anaphylaxis, Kounis syndrome; CAD = coronary artery disease; HF = heart failure; KS = Kounis syndrome; LGE = late gadolinium enhancement; LVEF = left ventricular ejection fraction; RWMA = regional wall motion abnormalities; TTC = Takotsubo cardiomyopathy.

### Future directions

Cardiovascular multimodal imaging and histological assessment are central to the diagnostic and therapeutic framework of the ATAK complex. Multimodal imaging is key to advanced phenotyping of TTC, KS, and eosinophilic myocarditis, while EMB remains pivotal to define the underlying substrate. In our case, histological findings were consistent with vasculitis without evidence of myocarditis, while molecular analysis excluded an infectious etiology, providing key guidance for immunosuppressive therapy. Early identification of these features is critical, as high-dose corticosteroids have been associated with reduced in-hospital mortality in inflammatory myocardial conditions.^35^ We propose a diagnostic and therapeutic algorithm (Figure 6) that integrates multimodal imaging and EMB for early diagnosis and guidance to timely initiation of targeted therapies across the ATAK spectrum. The absence of randomized clinical trials and the limited availability of high-quality data preclude strong class recommendations. In TTC, given the transient nature of the disease, therapy should primarily be supportive. In low-risk cases, a heart failure–oriented strategy can be adopted, with early discharge considered in patients with LVEF >45% and no complications after reassessment. High-risk patients require admission with continuous electrocardiographic monitoring for at least 72 hours, withdrawal of sympathomimetic agents, optimization of heart failure therapy, and consideration of noncatecholaminergic inodilators or mechanical circulatory support.^13^ In KS, management should follow ACS guidelines,^36^ with adjunctive corticosteroids^37^^,^^38^ and H1/H2 antihistamines (eg, diphenhydramine, ranitidine) to control allergic manifestations.^20^ In patients with distributive or mixed cardiogenic-distributive shock, fluid resuscitation is indicated, with cautious use of catecholamines, recognizing that epinephrine remains the first-line lifesaving therapy in anaphylaxis, whereas antihistamines and glucocorticoids are not.^39^ Calcium-channel blockers and nitrates can be beneficial in hypersensitivity-related vasospasm. Management of eosinophilic myocarditis depends on clinical severity (eg, cardiogenic shock, reduced LVEF, malignant arrhythmias) and feasibility of EMB. In this setting, early initiation of high-dose corticosteroids is recommended and associated with improved outcomes.^35^

**Figure 6:**
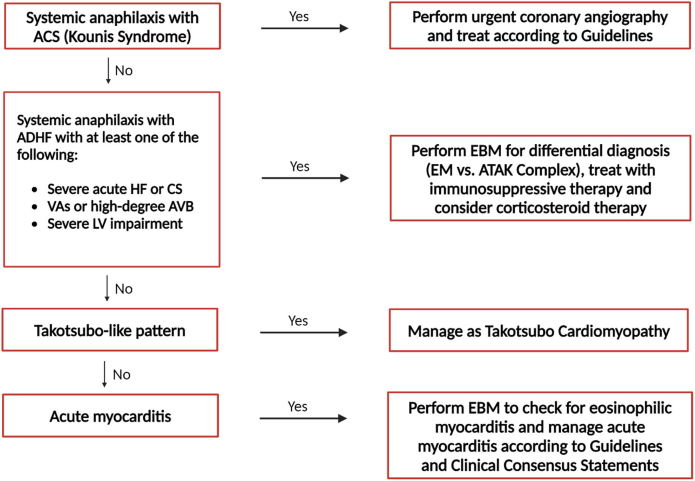
Proposed Flow Chart for Systematic Approach to Anaphylaxis With Cardiac Involvement ACS = acute coronary syndrome; ADHF = acute decompensated heart failure; HF = heart failure; ATAK = adrenaline, Takotsubo cardiomyopathy, anaphylaxis, Kounis syndrome; AVB = atrioventricular block; CS = coronary syndrome; EBM = endomyocardial biopsy; EM = eosinophilic myocarditis; LV = left ventricular; VA = ventricular arrhythmia.

## Conclusions

The ATAK complex embodies the convergence of allergic, vascular, and myocardial injury under catecholaminergic stress. It reflects a dynamic continuum in which exogenous and endogenous triggers provoke coronary vasospasm, endothelial dysfunction, and microvascular inflammation, potentially culminating in Takotsubo cardiomyopathy. The case presented in this mini-review, supported by histological evidence of coronary vasculitis, underscores the central role of inflammation in the pathogenesis of this syndrome. Timely recognition through multimodal imaging, early endomyocardial biopsy, and institution of corticosteroid therapy is crucial, especially in the presence of hemodynamic instability and evolving myocardial dysfunction. Given the rarity and heterogeneous clinical and pathophysiological presentation of the ATAK complex, further prospective studies are warranted to define its incidence, improve risk stratification, and refine therapeutic pathways.

## Funding Support and Author Disclosures

Dr Ricci was supported by the European Union—Next Generation EU, under the National Recovery and Resilience Plan (NRRP), Mission 4 Component 2—M4C2, Investment 1.5—Call No. 3277 of 30.12.2021—The Italian Ministry of University and Research (MUR), Award Number: ECS00000041, project title: “Innovation, digitalization and sustainability for the diffused economy in Central Italy,” Concession Degree No. 1057 of 23.06.2022 adopted by the Italian Ministry of University and Research (MUR). CUP: D73C22000840006. All other authors have reported that they have no relationships relevant to the contents of this paper to disclose.

## References

[1] Dworetzky M., Cohen S., Cohen S.G., Zelaya-Quesada M. (2002). Portier, Richet, and the discovery of anaphylaxis: a centennial. J Allergy Clin Immunol.

[2] Pühringer V., Jilma B., Herkner H. (2023). Population-based incidence of all-cause anaphylaxis and its development over time: a systematic review and meta-analysis. Front Allergy.

[3] Sampson H.A., Muñoz-Furlong A., Campbell R.L. (2006). Second symposium on the definition and management of anaphylaxis: summary report—Second National Institute of Allergy and Infectious Disease/Food Allergy and Anaphylaxis Network symposium. J Allergy Clin Immunol.

[4] Neugut A.I., Ghatak A.T., Miller R.L. (2001). Anaphylaxis in the United States. Arch Intern Med.

[5] Golden D.B. (2007). What is anaphylaxis?. Curr Opin Allergy Clin Immunol.

[6] Marone G., Genovese A., Varricchi G., Granata F. (2014). Human heart as a shock organ in anaphylaxis. Allergo J Int.

[7] Marone G., Patella V., de Crescenzo G., Genovese A., Adt M. (1995). Human heart mast cells in anaphylaxis and cardiovascular disease. Int Arch Allergy Immunol.

[8] Marone G., Triggiani M., Depaulis A. (2005). Mast cells and basophils: friends as well as foes in bronchial asthma?. Trends Immunol.

[9] Kaartinen M., Penttilä A., Kovanen P.T. (1994). Accumulation of activated mast cells in the shoulder region of human coronary atheroma, the predilection site of atheromatous rupture. Circulation.

[10] Patella V., Marinò I., Lampärter B., Arbustini E., Adt M., Marone G. (1995). Human heart mast cells. Isolation, purification, ultrastructure, and immunologic characterization. J Immunol.

[11] Mackins C.J. (2006). Cardiac mast cell-derived renin promotes local angiotensin formation, norepinephrine release, and arrhythmias in ischemia/reperfusion. J Clin Invest.

[12] Triggiani M., Patella V., Staiano R.I., Granata F., Marone G. (2008). Allergy and the cardiovascular system. Clin Exp Immunol.

[13] Lyon A.R., Bossone E., Schneider B. (2016). Current state of knowledge on Takotsubo syndrome: a position statement from the taskforce on Takotsubo syndrome of the heart failure Association of the European Society of Cardiology. Eur J Heart Fail.

[14] Arcari L., Limite L.R., Autore C., Volpe M., Musumeci M.B. (2018). A unique case of secondary Takotsubo syndrome. G Ital Cardiol (Rome).

[15] Lyon A.R., Citro R., Schneider B. (2021). Pathophysiology of Takotsubo syndrome. J Am Coll Cardiol.

[16] Khan H., Gamble D.T., Rudd A. (2023). Structural and functional brain changes in acute Takotsubo syndrome. JACC Heart Fail.

[17] Horowitz J.D., Nguyen T.H. (2022). Takotsubo syndrome. JACC Cardiovasc Imaging.

[18] Wilson H.M., Cheyne L., Brown P.A.J. (2018). Characterization of the myocardial inflammatory response in acute stress-induced (Takotsubo) Cardiomyopathy. JACC Basic Transl Sci.

[19] Kounis N.G., Zavras G.M. (1991). Histamine-induced coronary artery spasm: the concept of allergic angina. Int J Clin Pract.

[20] Abdelghany M., Subedi R., Shah S., Kozman H. (2017). Kounis syndrome: a review article on epidemiology, diagnostic findings, management and complications of allergic acute coronary syndrome. Int J Cardiol.

[21] Kounis N.G., Koniari I., Velissaris D., Tzanis G., Hahalis G. (2019). Kounis syndrome—not a single-organ arterial disorder but a multisystem and multidisciplinary disease. Balkan Med J.

[22] Gilfillan A.M., Tkaczyk C. (2006). Integrated signalling pathways for mast-cell activation. Nat Rev Immunol.

[23] Culpepper O., Jerger M., Belen S. (2018). Allergic angina: a new addition to the differential diagnosis and pathophysiology of chest pain. J Am Coll Cardiol.

[24] Kounis N.G., Filippatos G.S. (2007). Takotsubo and Kounis syndrome: is there any association?. Circ J.

[25] Kounis N.G., Mplani V., de Gregorio C., Koniari I. (2023). Attack the ATAK; A challenging contemporary complex: pathophysiologic, therapeutic, and preventive considerations. Balkan Med J.

[26] Singh G., Manickam A., Rathod R.C. (2015). Takotsubo cardiomyopathy in a patient with pituitary adenoma and secondary adrenal insufficiency. Indian J Crit Care Med.

[27] Gicquel-Schlemmer B., Beller J.P., Mchalwat A., Gicquel P. (2015). Fatal Takotsubo cardiomyopathy due to epinephrine in shoulder arthroscopy. Orthopaedics Traumatol Surg Res.

[28] Mustehsan M.H., Jahufar F., Arora S. (2019). A diagnostically challenging infusion reaction—Kounis, Takotsubo, or the ATAK!. JAMA Intern Med.

[29] Margonato D., Abete R., Di Giovine G. (2019). Takotsubo cardiomyopathy associated with Kounis syndrome: a clinical case of the “ATAK complex.”. J Cardiol Cases.

[30] Ballesteros R.V., Polo J.C.G., Olmos C., Vilacosta I. (2023). Kounis and Takotsubo, two syndromes bound by adrenaline: the “ATAK” complex. Case Rep Cardiol.

[31] Minciullo P.L., Amato G., Vita F., Pioggia G., Gangemi S. (2023). ATAK complex (adrenaline, Takotsubo, anaphylaxis, and Kounis hypersensitivity-associated coronary syndrome) after COVID-19 vaccination and review of the literature. Vaccines (Basel).

[32] Alarcón Gallardo E., Escudero Apesteguía R., Sanz Bescós C. (2024). ATAK complex due to amoxicillin: a case report. J Investig Allergol Clin Immunol.

[33] Li S., Ding P., Wang C., Long K., Gao P. (2024). ATAK complex (adrenaline, Takotsubo, anaphylaxis, and Kounis hypersensitivity-associated coronary syndrome) related to latamoxef administration—a case report. Front Cardiovasc Med.

[34] Puri P., Kachhadia M.P., Sardana P., Bhagat R., Dekowski S.S., Fohle E. (2024). Adrenaline, Takotsubo, anaphylaxis, and Kounis syndrome (ATAK) complex unveiled: integrating Takotsubo and Kounis syndromes in the context of chemotherapy-related anaphylaxis. Cureus.

[35] Byrne R.A., Rossello X., Coughlan J.J. (2023). 2023 ESC Guidelines for the management of acute coronary syndromes. Eur Heart J.

[36] Fassio F., Losappio L., Antolin-Amerigo D. (2016). Kounis syndrome: a concise review with focus on management. Eur J Intern Med.

[37] Takagi S., Goto Y., Hirose E. (2004). Successful treatment of refractory vasospastic angina with corticosteroids-coronary arterial hyperreactivity caused by local inflammation?. Circ J.

[38] Ioannidis T.I., Mazarakis A., Notaras S.P. (2007). Hymenoptera sting-induced Kounis syndrome: effects of aspirin and β-blocker administration. Int J Cardiol.

[39] Ammirati E., Cipriani M., Lilliu M. (2017). Survival and left ventricular function changes in fulminant versus nonfulminant acute myocarditis. Circulation.

